# Reconstruction of an ancestral *Yersinia pestis *genome and comparison with an ancient sequence

**DOI:** 10.1186/1471-2164-16-S10-S9

**Published:** 2015-10-02

**Authors:** Wandrille Duchemin, Vincent Daubin, Eric Tannier

**Affiliations:** 1 Laboratoire de Biométrie et Biologie Évolutive, LBBE, UMR CNRS 5558, University of Lyon 1, 43 boulevard du 11 novembre 1918, 69622 Villeurbanne, France; 2 Institut National de Recherche en Informatique et en Automatique (INRIA) Grenoble Rhône-Alpes, 655 avenue de l'Europe, 38330 Montbonnot, France

**Keywords:** phylogeny, rearrangements, plague, molecular evolution

## Abstract

**Background:**

We propose the computational reconstruction of a whole bacterial ancestral genome at the nucleotide scale, and its validation by a sequence of ancient DNA. This rare possibility is offered by an ancient sequence of the late middle ages plague agent. It has been hypothesized to be ancestral to extant *Yersinia pestis *strains based on the pattern of nucleotide substitutions. But the dynamics of indels, duplications, insertion sequences and rearrangements has impacted all genomes much more than the substitution process, which makes the ancestral reconstruction task challenging.

**Results:**

We use a set of gene families from 13 *Yersinia *species, construct reconciled phylogenies for all of them, and determine gene orders in ancestral species. Gene trees integrate information from the sequence, the species tree and gene order. We reconstruct ancestral sequences for ancestral genic and intergenic regions, providing nearly a complete genome sequence for the ancestor, containing a chromosome and three plasmids.

**Conclusion:**

The comparison of the ancestral and ancient sequences provides a unique opportunity to assess the quality of ancestral genome reconstruction methods. But the quality of the sequencing and assembly of the ancient sequence can also be questioned by this comparison.

## Background

Extant species are derived from a process of evolution and diversification from species now disappeared. These species are called ancient in general and ancestral if they left a descendant. Ancestral genomic sequences can be estimated through computation from a set of extant sequences related by a phylogeny and a model of evolution [1], while ancient genomic sequences in general can be sequenced from the remains of dead organisms [2].

### Ancestral genome reconstruction

Ancestral genome reconstruction can consist in predicting a gene content in ancestral species [3], and for each gene its sequence [1]. While originally used to study proteins or isolated genes, ancestral genome reconstructions are now robust at a scale larger than the gene, for fragments where no rearrangement have occurred [4]. Methods for inferring ancestral gene orders have also been explored [5-8]. Together, these methods open the way to the reconstruction of complete ancestral genomes, including their sequences.

Obtaining ancestral sequences can allow, through the study of physical properties of the reconstructed molecules, the inference of the paleoenvironnements in which these molecules evolved [9]. These methods also allow access to an oriented and ordered view of molecular events along the history of life. Moreover, they offer a better understanding of this history and can further our knowledge of the mechanisms linking organic sequences to their functions [10].

Despite this, ancestral sequence reconstruction suffers from several limits. Along with the study of molecular evolution, it relies on the validity of models and their fundamental hypothesis. Furthermore, given that we are interested in a phenomenon often distant in time, it is at best difficult to obtain proofs validating proposed predictions. Thus, the validation of ancestral reconstruction methods is often limited to robustness tests, or simulations that themselves rely on the validity of the models of evolution [1].

### Ancient genome sequencing

Ancient DNA sequences is another way to have an access to the past history of living organisms. Under certain conditions it is possible to obtain genetic material through the sequencing of the remains of an organism. Ancient DNA sequencing began in the middle of the 80s with the cloning and sequencing of fragments of mitochondrial DNA in a museum specimen of *Equus quagga*, an extinct equine species that disappeared in the XIX*^th ^*century [11]. The advent of PCR methods [12] and high-throughput sequencing [13] followed by what is called third generation sequencing [14] allowed the sequencing of several extinct animals [15-17], ancient unicellular eukaryotes [18,19], bacteria [2,20,21], metagenome [22], or virome [23].

The ancient sequences disclose a new source of information concerning the evolution of lineages of interest. They have already been used, among other things, to understand the dynamic of extant populations of the genus *Homo *[24-26], or other animals [27], to correct and recalibrate phylogenies [17], or to better understand past pandemics [18,2,21].

However, along with the problems specific to sequencing technologies, ancient DNA sequencing is limited by the post-mortem chemical degradation of DNA molecules throughout time. Thus, like fossils, ancient sequences are scarce while, unlike them, limited to recent times.

### *Yersinia pestis*

**Figure 1:**
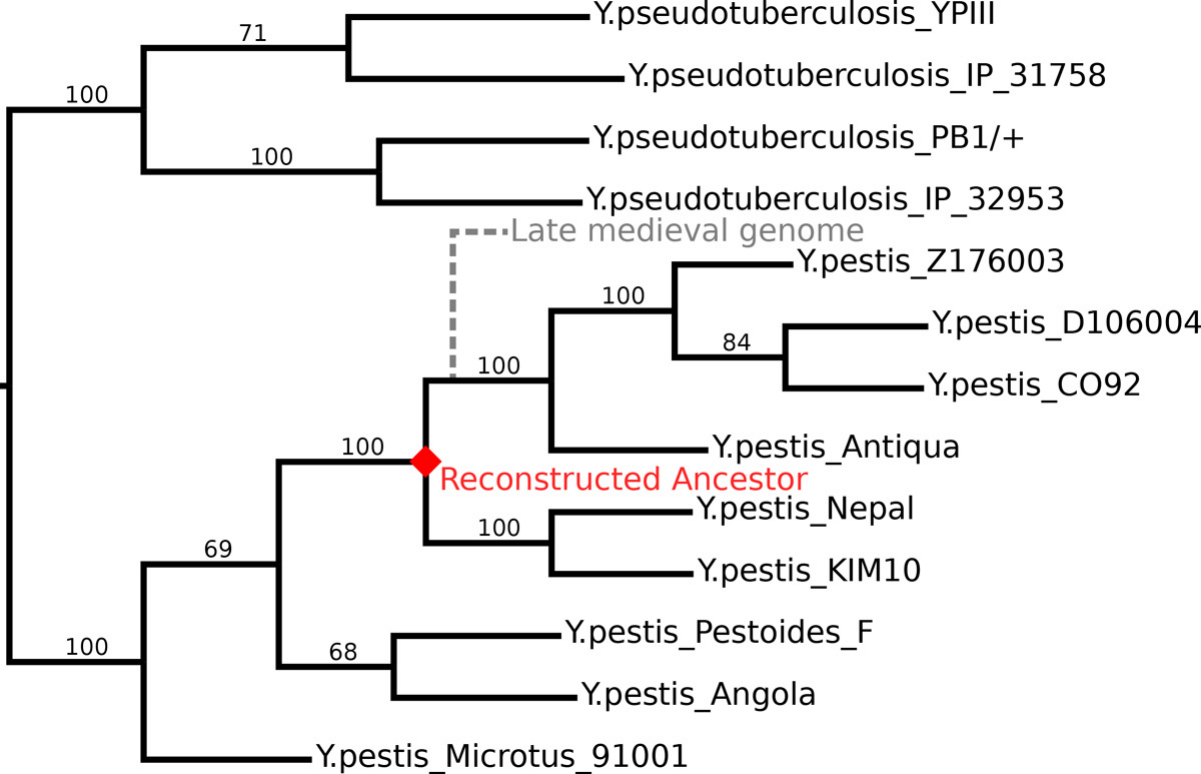
**Yersinia pestis and pseudotuberculosis phylogeny**. Tree obtained using a 1971 universal gene families concatenate. Bootstrap values are figured on the branches. For readability, the figured branch length is the inverse of the ten-logarithm of the real branch-length. The ancestral species of interest to us is figured as a red diamond. The late medieval ancient genome hypothetical position is figured in gray and dashed.

Classified among *Enterobacteriaceae, Yersinia pestis *is the bacterium thought to be responsible for the bubonic plague and the pneumonic plague. It diverged from the *Yersinia pseudotuberculosis *lineage, in part through the acquisition of two plasmids [28]. It has been demonstrated that strains of *Yersinia pestis *caused the black death of 1347-1353 AD that is thought to have killed between a third and half of the European population at that time and persisted in Europe until the middle of the XVIII*^th ^*century [29]. An ancient genome has been extracted and sequenced [2]. It was the first whole ancient bacterial genome. Based on a substitution pattern compared to extant *Yersinia *species, it has been hypothesized to take place on the extant species phylogeny in the vicinity of a known speciation node leading to two set of extant, sequenced and annotated strains of the bacterium (see Figure 1).

**Figure 2:**
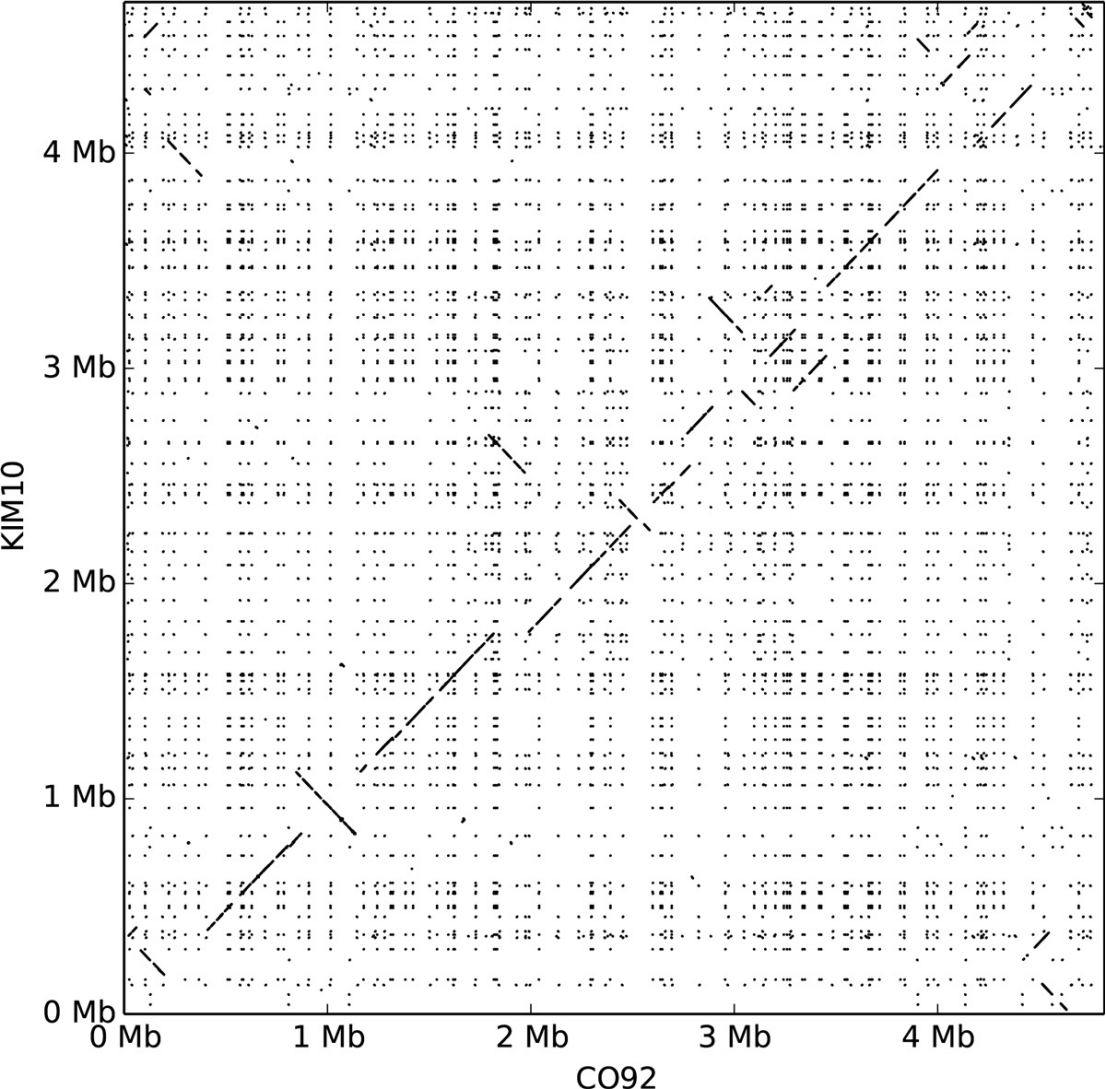
**Dotplot between the sequence of two extant strains of Yersinia pestis: CO92 and KM10**. Both strains are descendants of the ancestor we focus on. Data was obtained by aligning the sequence of strain KIM10 on the sequence of strain CO92 using megablast (default parameters, only hits with a length *>*10^2.5 ^were kept).

The existence of several sequenced and annotated extant genomes as well as the relatively short evolutionary time separating them make their ancestor a good candidate for an ancestral reconstruction including both sequence and gene organization along the chromosome and the plasmids. However despite the short evolutionary time, while substitutions are quite rare [2], there is a very active dynamics of rearrangements, insertion sequences propagation, duplications, copy number variation (see Figure 2), which makes the problem challenging.

The late-medieval ancient genome, likely close to that ancestor, offers a validation opportunity for the ancestral reconstruction method. We achieve here this reconstruction and perform the comparison.

Note that a sequence of the same genome was proposed recently by Rajaraman et al. [30], but was not issued from ancestral reconstruction. The contigs of the ancient genome were scaffolded with a method including the phylogeny of relatives, and some parts of the assembly could be corrected, but what we present here is not using at all the ancient sequence in the reconstruction phase, it is done only from independent extant data.

## Methods

**Figure 3:**
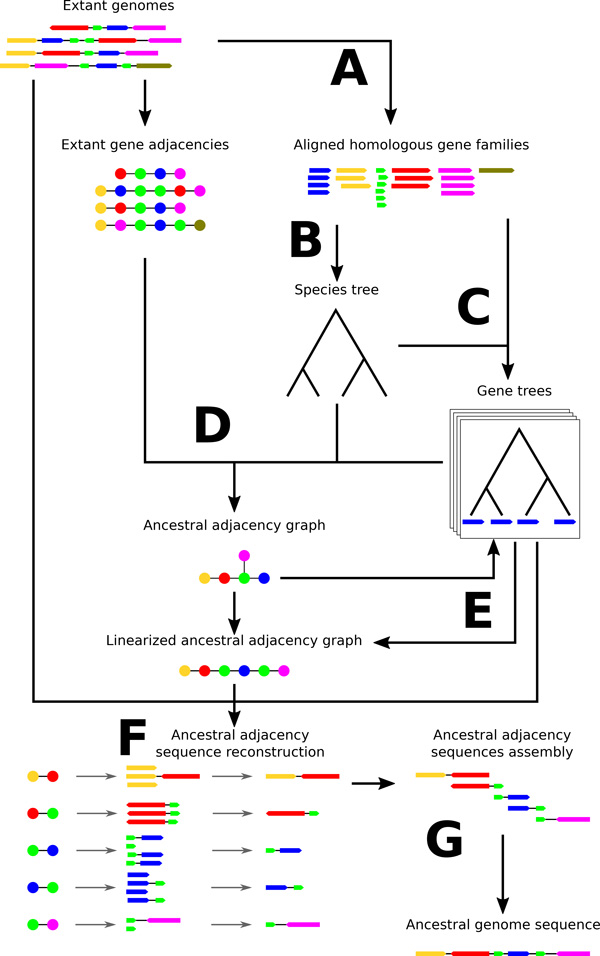
**Protocol used to obtain the ancestral gene order and sequence of a Yersinia pestis ancestor**. A) Extraction and filtering of gene families from extant genomes and alignment. B) Reconstruction of the species tree using a concatenate of the variant positions of 1971 universal gene families. C) ML reconstruction of gene trees followed by the collapse of any non-supported branch (bootstrap *<*99) and the resolution of the created polytomies using the species tree as a guide. D) Inference of ancestral gene adjacencies using DeCo. E) Detection and correction of wrongly inferred gene trees based on the ancestral adjacency graph linearity. F) Reconstruction of the ancestral sequence of each gene adjacency from their extant descendants. G) Alignment of the consecutive ancestral adjacency sequences to assemble the ancestral genome. Similar colors indicates homology. Dots represent a gene as a node in an adjacency graph while oriented segments represent a gene as a sequence.

An overview of the method, including species tree construction, gene tree construction and reconciliation, gene order inference and gene tree corrections according to this gene order, and eventually genic and intergenic sequence prediction, is illustrated on Figure 3.

### Data set

The data consists in 13 *Yersinia *annotated genomes (Figure 1) from which we extract 3772 homologous protein gene families containing at least two genes, using the HOGENOM database [31]. Of these, 1971 have exactly one copy per extant strain. This step corresponds to part A in Figure 3.

### Species tree

Using Muscle [32] (default parameters), we aligned the 1971 families, concatenated the variable sites of all alignments and obtained a phylogenetic tree using PhyML [33] (100 bootstraps, otherwise default parameters) that we rooted by separating the *pestis *from the *pseudotuberculosis *clades, according to a consensus in the literature. In our tree the branch separating the two clades is well supported, as well as the branches surrounding the ancestor that we wish to reconstruct (see Figure 1). This step corresponds to part B in Figure 3.

### Gene trees

All gene families sequences were then aligned using Prank [34] and one gene tree per family was computed using PhyML (100 bootstraps, otherwise default parameters). Because we are aligning recently diverged strains of the same organisms [35], the sequences often have not diverged enough to allow an unambiguous tree reconstruction. So we collapsed all branches with a support lower than 99 and then used ProfileNJ [36] to solve the created polytomies. ProfileNJ reconstructs species tree branches instead of collapsed branches and chooses among several solutions with a Neighbor-Joining formula. Distances for the Neighbor-Joining part were computed with bppdist, a Bio++ suite software [37] (GTR + Γ(4) model).

ProfileNJ also roots the gene trees according to "Last Common Ancestor" reconciliation method, annotating internal nodes with duplications or speciations, and choosing a root minimizing the number of duplications.

Reconciled gene trees depict the history of the gene family, including all ancestral genes, uniquely defined by the reconciliation.

This step corresponds to part C in Figure 3.

### Gene families filtering

From the 3772 gene families, some were discarded because they showed signal of a process that we do not handle well in our pipeline, gene transfer. Transfer was suspected when a branch in the reconciled gene tree would correspond to at least 4 independent losses in the species tree. We also removed the families with more than 5 genes in the black death ancestor, suspecting insertion sequences, which are poorly handled by the method. We also removed families containing genes fully included in other genes: as we model the evolution of gene orders, these would be difficult to handle. We eventually removed families when the reconciled gene tree did not contain a gene in the ancestor we want to reconstruct.

The final data set contained 3656 families. Note that when removing gene families from the study, we do not necessarily give up the reconstruction of parts of the ancestral sequence. We just define the removed parts as intergenic. As we also reconstruct intergenic sequences, this simply modifies the resolution at which we are able to detect rearrangements.

### Extant gene order and adjacencies

Each gene is a segment of a chromosome or a plasmid and has a start and an end position on it. We identify these positions as the *extremities *of the gene. A start position may be greater than an end position: the order of the extremities defines the *orientation *of the gene. We model each genome by a graph, whose nodes are gene extremities of genes in that genome. We put an edge, called an *adjacency *between pairs of extremities of a same gene. Additionally if genes *AA′* and *BB′* are consecutive (*A *and *A′* are the extremities of the first gene, appearing in that order on the chromosome or plasmid, and *B*, *B′* are the extremities of the second gene), we put an adjacency between *A′* and *B*. So extant genomes are sets of disjoints cycles in a graph, modeling chromosomes and plasmids.

Gene extremities can be clustered into families, inherited from gene families, and also inherit the reconciled gene tree.

### Ancestral gene order

Ancestral adjacencies between gene extremities were inferred using DeCo [7]. It models the evolution of an adjacency between two gene extremities following a parsimony principle, *i.e.* minimizing the number of gains and breakages of adjacencies, due to rearrangements. It takes as input the species tree, all gene trees, and extant adjacencies, and proposes a set of ancestral adjacencies between ancestral gene extremities defined by the reconciled gene trees. This step corresponds to part D in Figure 3.

**Figure 4:**
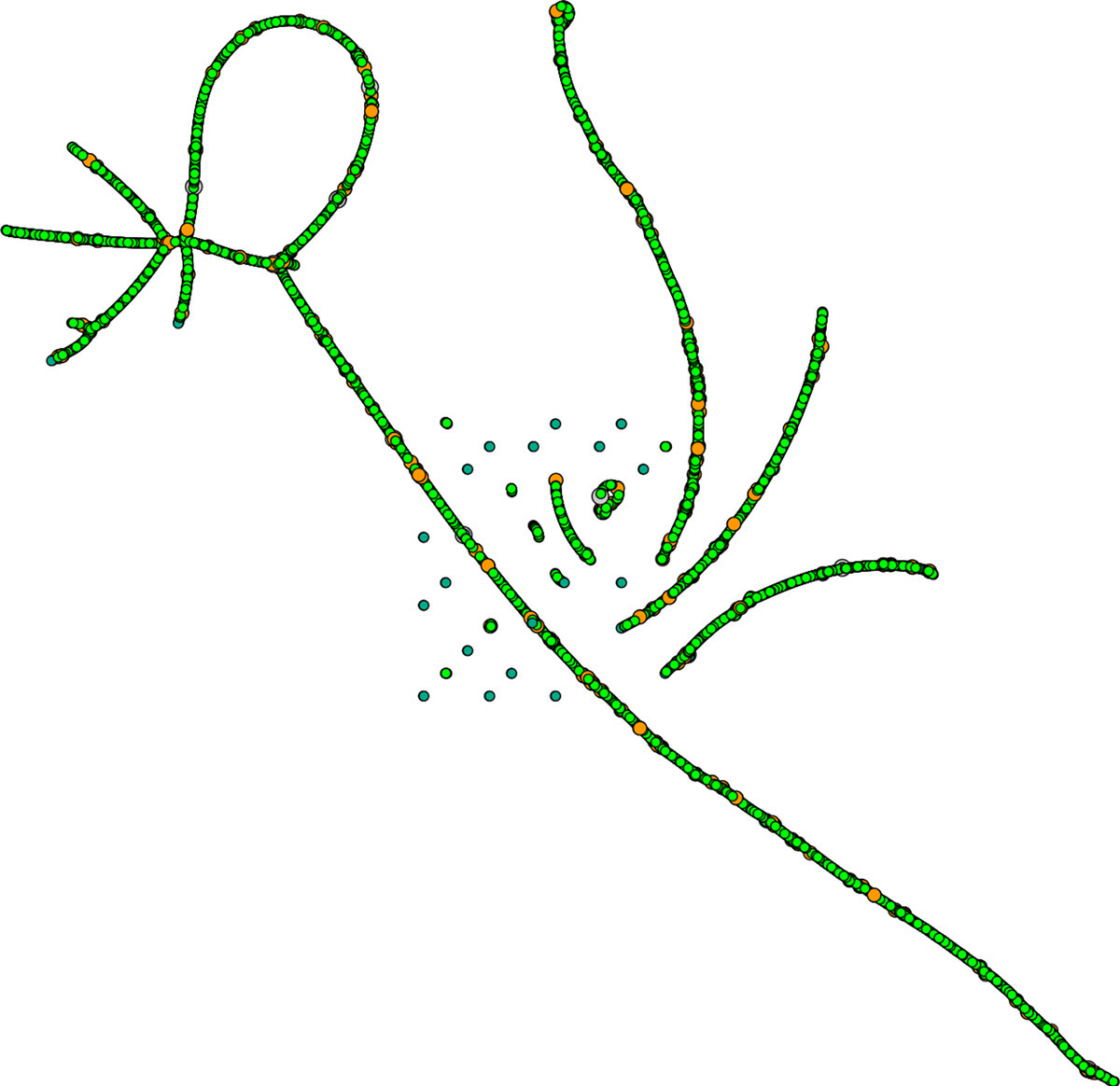
**Ancestral adjacency graph obtained using DeCo on the set of 3656 gene families**. Each node is colored according to its number of neighbors: green for two (ideal, linear case), turquoise for one (where one adjacency has been lost), orange for three and gray for four (when an error in the number of ancestral copies creates conflict in the ancestral gene order).

DeCo assumes that adjacencies evolve independently. This means in particular that ancestral gene extremities can be involved in an arbitrary number of adjacencies. Ancestral gene extremities and adjacencies are not necessarily made of cycles as extant genomes, so we call this object an *adjacency graph*. Figure 4 shows the obtained adjacency graph at this step. While most of it shows a linear or circular structure, there are some gene extremities with too many adjacencies, others with not enough.

There can be several reasons for the adjacency graph not to be a collection of paths and cycles, as we would expect if the data and methods were perfect. Incorrect gene trees are probably the major source of such discrepancies, while others may come from uncertainties in adjacency history inference.

We transform the adjacency graph into a genome (*i.e*. an adjacency graph that is a collection of paths and cycles), first by correcting gene trees, by operations we call zipping and unzipping, then by removing a minimum number of adjacencies so that the remaining graph is a genome.

### Correcting gene trees

**Figure 5:**
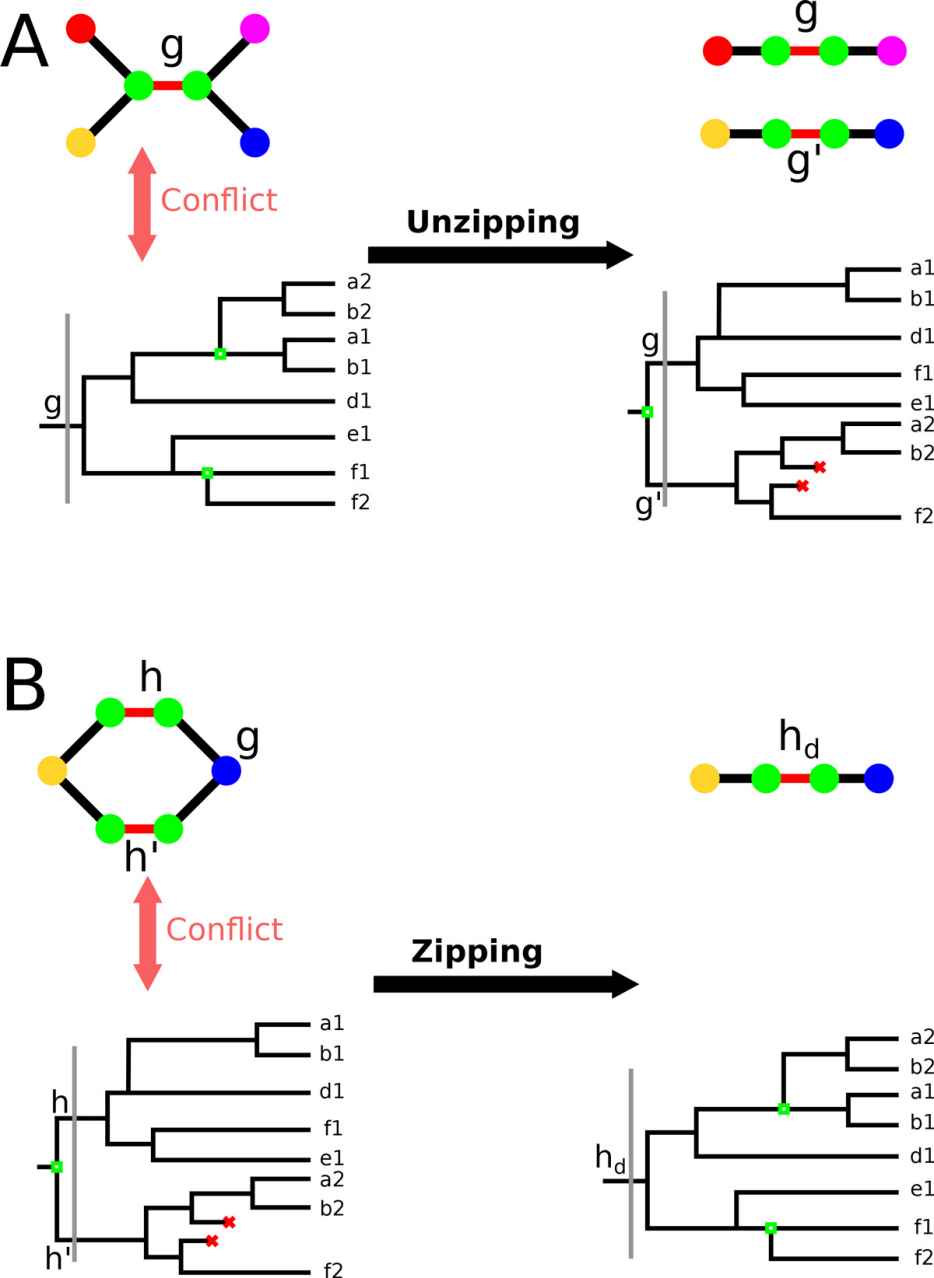
**Illustration of the unzipping and zipping on gene trees and adjacency graphs**. A) Prior to linearization (left of the black arrow), the gene *g *exists in one copy in the ancestor (vertical gray line on the tree) and two independent duplications occurs in its descendants (green hollow squares). In the ancestral adjacency graph above each of *g *extremities displays two neighbors. Unzipping (right of the black arrow) modifies the tree so that there are two ancestral copies *g *and *g′* each corresponding to a different path in the ancestral adjacency graph (losses in the tree are displayed as red crosses). B) Prior to linearization (left of the black arrow), two ancestral copies of the same gene *h *and *h′* exist in the ancestor (vertical gray line on the tree; losses in the tree are displayed as red crosses). In the ancestral adjacency graph above the extremities of *h *and *h′* each share a neighbor, forming a non-linear pattern. Zipping (right of the black arrow) modifies the tree so that there only one ancestral copy *h_d _*followed by independent duplications in its descendants (green hollowed squares), forming one linear path in the ancestral adjacency graph.

This step corresponds to part E in Figure 3 and a more detailed picture is on Figure 5.

#### Unzipping

Each ancestral gene extremity of a gene *g *should have at most two adjacencies. If one has more than two, a first hypothesis can be that in the real ancestral genome, the gene *g *was duplicated in two copies, and each copy would carry some of the adjacencies of *g*.

If in one extant species, there are two homologous copies of the gene *g*, and their extremities share the homologs of the adjacencies attributed to an extremity of *g*, then we perform the *unzipping *operation.

It consists in making two genes out of *g* by modifying the gene tree *T *of the gene family containing *g*. Only the subtree rooted at *g *is changed, into a subtree rooted at a new duplication node with two descendants: *g *and a new gene *g′*. Then the two subtrees rooted at *g *and *g′* are reconstructed, first by assigning all leaves to *g *or *g′* according to their neighborhood; Then by constructing subtrees on these leaves using ProfileNJ. In the case where some leaves can't be assigned to either *g *or *g′* using their neighborhood (*i.e*. their extant neighbors are not descendant of any of the ancestral neighbors), then leaves are assigned to one of the two set of leaves according to their mean phylogenetic distances with them. Where there is a tie (for instance if all sequences are identical, all distances are null), the leaf is randomly assigned to one of the two leaf-set.

Figure 5A gives an example of an unzipping operation on the ancestral adjacency graph and on the gene tree.

If the unzipping procedure increases the number of adjacencies incident to a gene extremity of a gene *h *in the immediate neighborhood of *g *in the adjacency graph, then the unzipping procedure is applied to *h *as well, and then to its neighbors, until the region is linearized.

#### Zipping

Another possible reason for a gene *g *to be involved in more than two adjacencies is that two of these adjacencies *gh *and *gh′* concern two paralogs *h *and *h′* which in reality should form only one gene. In that case we perform a *zipping *operation, similar to the one described in [38].

Let *h_d _*be the last common ancestor of *h *and *h′* in their gene tree. Suppose it is assigned to species *s*, whose descendants are *s*_1 _and *s*_2_. It is a duplication node, and we turn it into a speciation node by giving it two descendant nodes *h*_1 _and *h*_2_, and assigning its descendant leaves to either one of them, depending on whether they are genes from descendants of *s*1 or *s*_2_. Then subtrees rooted at *h*_1 _and *h*_2 _are reconstructed using ProfileNJ.

Figure 5B gives an example of a zipping operation on the ancestral adjacency graph and on the gene tree.

Zipping produces a new ancestral gene *h_d _*instead of two paralogues *h *and *h′*. We propagate the same operation to the neighbors of the ancestral gene *h_d _*in the adjacency graph if they are themselves supernumerary paralogues.

Note that for zipping and unzipping, the propagation mechanism allows the treatment of several consecutive nodes, such that a large segmental duplication containing multiple genes can be dealt with as long as there exists a node to start the unzipping move (*e.g*. at one extremity of the segmental duplication).

#### Cutting

Zipping and unzipping are tested independently for each ancestral node with more than two neighbors. Each of them should decrease the number of gene extremities with more than two adjacencies. The operation that decreases it the most is kept.

If none of zipping and unzipping succeeds in removing all such supernumerary adjacencies (it is possible that none of the hypotheses applies), then we remove as few adjacencies as possible so that only gene extremities with at most two adjacencies remain. This is achieved using a maximum matching technique described in [39].

### Ancestral sequence reconstruction

Ancestral sequences have to be reconstructed by pieces, because they need a multiple alignment free of rearrangements. The pieces have to be glued together, and in order to avoid between pieces border problems, pieces have to overlap. This is why we reconstruct an ancestral sequence for all pairs of genes which are connected by an adjacency. Then pairs are aligned together on their common gene, and merged.

We orient each adjacent gene pair with a first and a second gene, each gene should be once the first gene of a pair, and once the second in another pair. We use the gene tree of the first gene as a guide, to construct a multiple sequence alignment with the extant sequences that contain this adjacent pair (thus, the sequences contains both genes and the sequence between them when they are neighbors in an extant species, and only the first gene of the adjacency when they aren't), and the ancestral sequence using Prank [34].

Gene sequences at the ends of contigs are reconstructed alone using their own tree. In consequence each inter-gene sequence is reconstructed once and each gene sequence is reconstructed twice and at least once with its own tree. We assemble the obtained ancestral sequences by aligning (using Smith & Waterman's algorithm) the ones sharing a gene and then making the consensus sequence of that alignment, favoring the sequence reconstructed with the tree of the aligned gene.

For instance, consider the ancestral path *ABC *(where *A,B *and *C *are genes), we reconstruct the ancestral sequence of *A *using its own tree, *AB *using *A*'s tree, *BC *using *B*'s tree and *C *using its own tree. Afterward the ancestral sequence of *A *is aligned with the ancestral sequence *AB*, favoring the sequence of *A *when computing the consensus. Then the sequence *AB *is aligned with the sequence *BC*, favoring the sequence *BC *in the consensus (as both sequences align on gene *B *and *BC *used *B*'s tree for the reconstruction). Finally, the sequence *ABC *is aligned with the sequence *C*, favoring *C *in the consensus.

A graphical view of these steps are given in Figure 3, parts F and G.

Note that, as stated before, the ancestral sequence reconstruction needs a multiple alignment free of rearrangements. This means that the size of the recombination events that can be taken into account for ancestral sequences reconstruction depends on the density of the markers (here, the gene extremities of 3656 gene families) used in the ancestral order reconstruction step.

## Results

### The shape of the ancestral genome

We perform the whole process of ancestral gene order reconstruction for three data sets: the whole set of filtered families, the set of D free families, without duplication and the DL free families, without duplication nor loss.

Ancestral gene order is computed with the whole set, but it gives fragmented paths in the adjacency graph. The fragments are progressively assembled using the D free and DL free gene orders.

The ancestral gene order was reconstructed for the chromosome (3342 genes) and the three plasmids (pCD: 74 genes, pMT: 87 genes, pPCP: 5 genes). The plasmids pCD and pPCP were obtained as circular elements in the adjacency graph, while the plasmid pMT was represented by one linear fragment. The chromosome was obtained as three linear components. To join these components, we ran DeCo on their six extremities using a gradient of adjacency gain/loss costs ratio (from 1/10 to 10/1) and scored each potential adjacency by the number of times it was observed. We then applied a weighted maximum matching technique [40] to extract the best possible order between the fragments (only one optimal solution remained).

**Figure 6:**
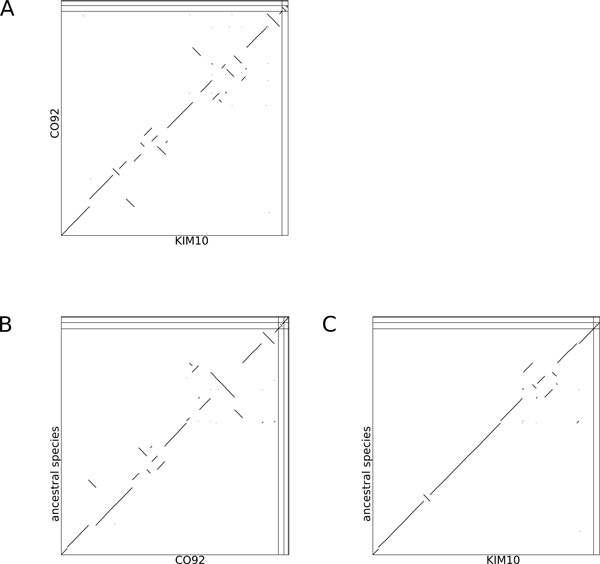
**Dotplot between the ancestral genome and two extant strains of Yersinia pestis: CO92 and KIM10**. Both strains are descendants of the ancestor we focus on. Data was obtained using the extant adjacency graphs of strains KIM10 and CO92 and concerns genes order. Vertical and horizontal lines separate the different molecules (here the chromosome and the plasmids). A) dotplot between the gene orders of the two extant strains KIM10 and CO92. B) dotplot between the gene orders of the ancestral genome and the extant strain CO92. C) dotplot between the gene orders of the ancestral genome and the extant strain KIM10.

The ancestral gene order is different from all extant genomes. For example it is an intermediary between the two extant strains *CO92 *and *KIM10*. Figure 6 B and C show the gene order comparison between the ancestral genome and two extant ones, while a comparison between the two extant ones is shown on Figure 6A. The isolated dots on the dotplots of Figure 6B and C are probably reconstruction errors. While they could be explained as small rearrangements, they probably are artifacts of the adjacency graph linearization method, like a leaf falsely associated to a subtree in an unzipping event for instance.

The ancestral sequences of the plasmids pCD, pMT and pPCP were entirely reconstructed, for a total of respectively 100.1 kb, 67.7 kb and 9.6 kb. Concerning the ancestral chromosome, a total of 4.7 Mb of ancestral sequence was reconstructed, which is close to the size of the extant chromosomes of *Yersinia pestis *strains (*e.g*. 4.7 Mb for the strain *Antiqua*). A lack of signal in extant genomes due to convergent rearrangements, prevented the reconstruction of four ancestral adjacencies. Because of these, the ancestral chromosome sequence is actually composed of four disjoint fragments (their sizes are respectively 3.44 Mb, 0.67 Mb, 0.40 Mb and 0.19 Mb).

The reconstructed ancestral sequences are avalaible in Additional file 1.

### Comparison to the ancient genome

Using Megablast [41] we aligned the 2134 ancient *Yersinia pestis *contigs obtained by Bos et al.[2] (avalaible at http://paleogenomics.irmacs.sfu.ca/FPSAC/, last accessed 19 june 2015) against the obtained ancestral genome, including chromosome and plasmids.

We examine 2179 hits of length *>*10^2.5^bp from 2087 contigs (see Additional file 2 for the bimodal distribution of hit lengths which justifies this threshold). The others are full of repeated elements, making the comparison difficult. As a consequence the examined hits all match to the chromosome and none to the plasmids.

#### Gene order

**Figure 7:**
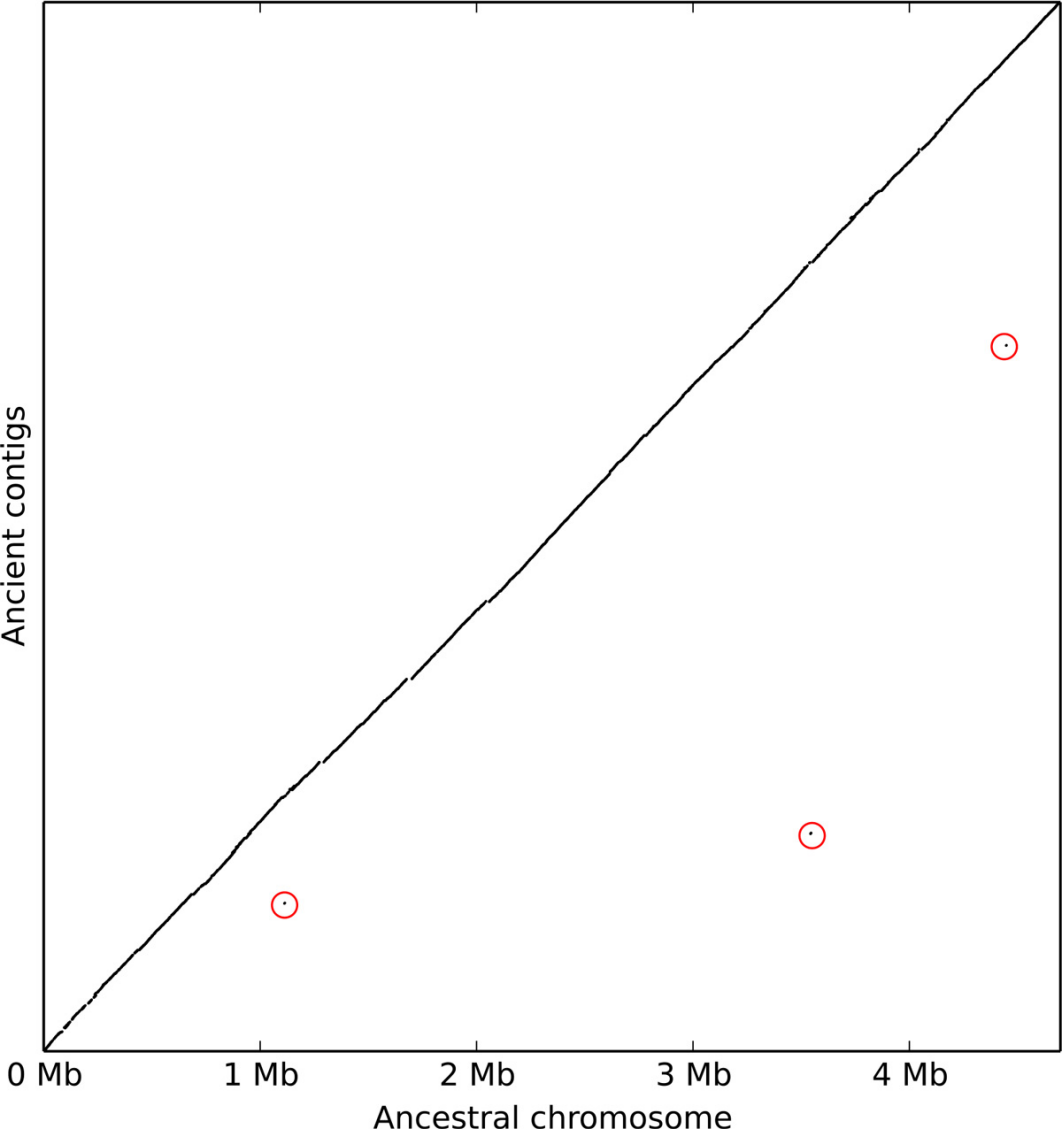
**Dotplot between the late medieval Yersinia pestis genomes and the reconstructed ancestral sequence**. The reconstructed chromosome was aligned to the 2134 ancient contigs, using megablast (default parameters, only hits with a length *>*10^2.5 ^were kept). Contigs are concatenated according to the reconstructed sequence, so the agreement is partly due to the fragmented nature of the ancient sequence. The contigs with hits departing from the diagonal are circled in red.

These hits show a quasi-total congruence between the organization of the ancient and ancestral sequence. Figure 7 represents the correspondence between the two in the form of a dotplot, where contigs of the ancient genome are concatenated according to the ancestral sequence. Three isolated dots deviate from the central line. Two of them concern large repeated regions, that is, the whole contigs match at several places. Only one seems to be a real discordance between the two genomes. Two contiguous regions of the contig hit on two different ancestral sequence fragments. This chimeric contig (number 8335 in [2]) had already been observed by Rajaraman et al. [30] in their scaffolding of the ancient genome. This stretches the proximity and the differences between the two approaches. Indeed, the latter, called FPSAC, takes as input the ancient contigs and the extant genomes, fragments the contigs according to their alignments to extant genomes, and orders fragments. Here we don't use at all the ancient contigs and start from extant genes. So we are independent of the extraction and assembly methodology for the ancient sequence, and we can compare to it. Moreover, all our sequences are computationally reconstructed, which was not the case of those obtained with FPSAC.

So at a large scale, there is only one difference which can be an assembly error in the ancient sequence or a derived mutation of the ancient bacteria, because the ancient configuration is not supported by extant genomes.

#### Sequences

At a finer scale, differences are more numerous. Approximately 81% of the 2084 contigs with a hit are exact matches to the ancestral genome. We examined some of the remaining and found that the differences could be explained by three kinds of error sources in the ancestral or ancient sequences:

• Lack of sufficient data for ancestral reconstruction: it is the case if only one of the two children which branches off the ancestor, in addition to an outgroup, support the presence of a sequence. In that case there is no comparison point to infer some bases, and some are inferred differently than in the ancient sequence.

• Lack of a good model of evolution at an intermediary scale, like duplication of small elements. They are here included in alignments and indel models, which do not account for repetitions.

• Assembly errors in the ancient sequence.

Consider for example the ancient contig number 497 where a mismatch occurs when aligned with the ancestral sequence. The mismatch is situated in an intergenic region of the ancestral genome that is present in one descendant of the reconstructed ancestor and two outgroup *Yersinia pestis *species. Consequently, the ancestral sequence was reconstructed using a tree where the node of interest was along a branch, missing a comparison point (*i.e*. another descendant) to choose between its descendant allele and the outgroup allele.

Consider also the ancient contig number 8849 which aligns with one mismatch to the reconstructed ancestor. At the position of the mismatch, all extant (group and outgroup species) sequences bear the same allele and thus the reconstructed ancestral sequence bears it too. However, the ancient contig bears another allele at that position. If we consider the ancient contig as correct, then this difference would be an original mutation on the ancient strain. Such an hypothesis could be checked by mapping the ancient reads to their contigs in order to assess the validity of that specific allele. However, we note that the original study [2] that used read data to call SNPs did not detect any that were specific to the ancient strain.

**Figure 8:**
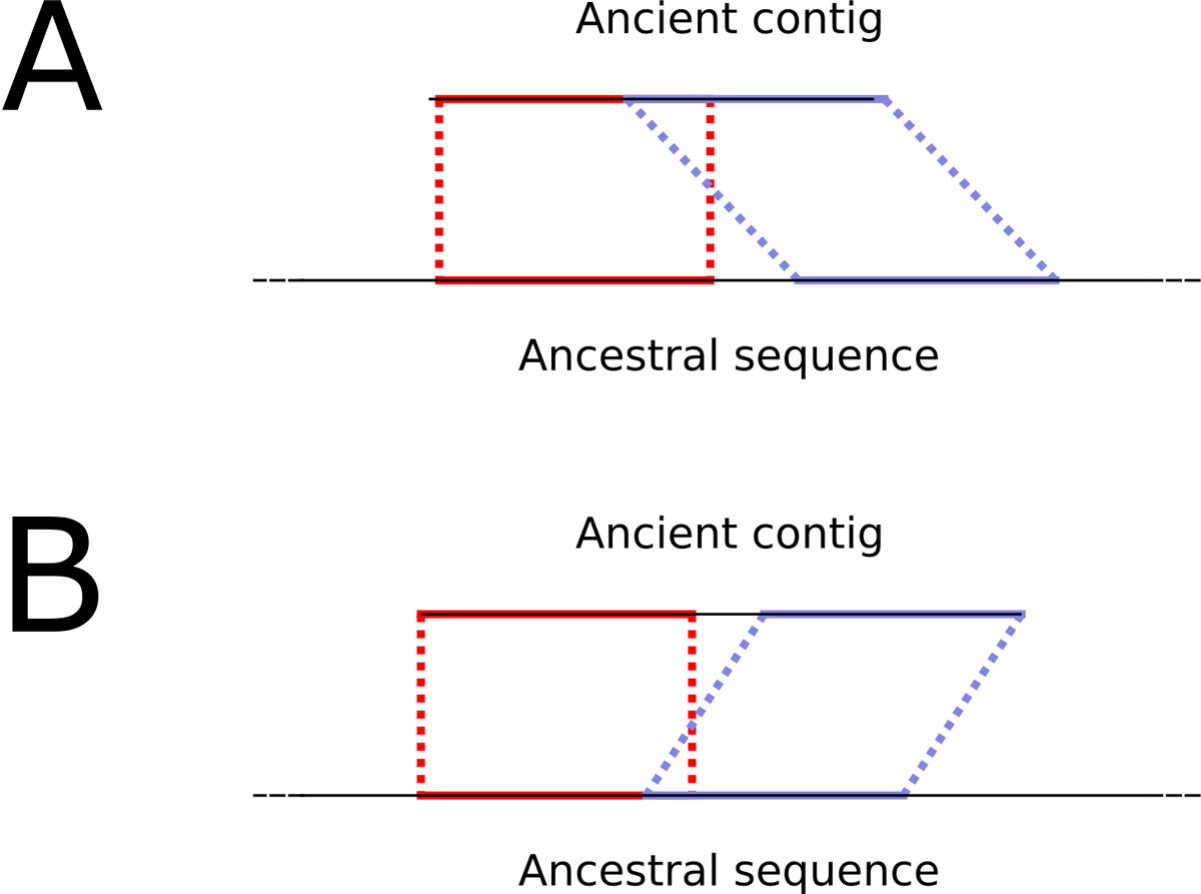
**Different hit patterns for ancient contigs on the ancestral sequence**. A) Contiguous or overlapping hits are more distant on the ancestor. B) Contiguous or distant hits are closer together (or overlapping) on the ancestor.

There are also differences that are more structural in kind. For example 43 contigs show some structural differences with the ancestral genome. On 39 of them, the ancient contig displays two contiguous or slightly overlapping hits that are more distant on the ancestral genome (on 21 occasions, they are more than 300 bp apart in the ancestral sequence), as in Figure 8A. On 4 ancient contigs, contiguous regions are shown as overlapping in the ancestral genomes, as in Figure 8B.

Such discrepancies can sometimes be explained by errors in the ancient sequence, especially in regions where repetitions occur. For instance, the case illustrated on Figure 8A, is seen on the contig number 8335 obtained by Bos et al.[2] (which is also the chimeric contig but this discrepancy is independent). Around position 1860, that ancient contig displays one occurrence of a 20-mer. However, the reconstructed ancestral sequence has two consecutive occurrences of that 20-mer. This region is situated in an intergenic region, so it has been reconstructed by an alignment of an adjacency with its two flanking genes. The extant species (descendant of the reconstructed ancestor or not) which have this gene adjacency all display two occurrences (in favor of the ancestral reconstruction) at the exception of *Yersinia pestis *strain CO92, the *Yersinia pestis *reference genome which was used to map the ancient reads in [2]. While the fact that we did not use the raw reads obtained in [2] prevents us to draw any definitive conclusion, this appears to be an error in the ancient sequence assembly, caused by a derived mutation in the genome used as a reference.

Conversely, it happens that similar patterns are better explained by errors in the reconstructed ancestral sequence. Such a case occurs on the locus where the ancient contig number 5613 maps. The situation is also similar to Figure 8A. Two contiguous regions hit at a distance of 1315 bp on the reconstructed ancestral sequence. The sequence separating the two hits in the ancestor is only supported by one extant descendant (*Nepal *strain) and the other extant descendants match the ancient contig in only one long hit. This seems to be an error due to the absence of an evolutionary model allowing big insertions. Prank models indels but 1315 bp is not really an indel but is rather an insertion of what should perhaps have been an evolutionary unit. It seems that the indel model prefers losing several times such a long DNA segment rather than inserting it once in a terminal branch of the phylogeny. So we can expect a small number of such false additions in the ancestral sequence.

## Discussion

A complete reconstruction of an ancestral genome at the nucleotide level requires to take into account evolutionary events at several scales: nucleotide substitutions, indels, duplications, losses, recombinations, transfers, transposable elements propagation, rearrangements. Each level is handled by dedicated bioinformatics tools which are rarely used together.

We associated here gene content/sequence/order tools in order to attempt the reconstruction of a whole ancestral bacterial genome, including a chromosome and three plasmids. We chose an organism from the *Yersinia pestis *clade because of a recently published ancient sequence. Despite being relatively recent at the evolutionary scale (650 years), the evolution at all levels, and in particular in genome structure and organization, makes the problem difficult. The difficulty can come from numerous events (rearrangements, insertion sequence dynamics), but also from scarce events (substitutions) that prevent reconstructing gene trees from sequences because of a lack of information.

We did not only assemble existing tools that handle evolution at different levels, but also report methodological novelties, like the zipping and unzipping processes to modify gene trees and linearize adjacency graphs. Using synteny information to construct gene trees is rarely achieved [36] and linearizing often only use cutting operations [39].

We cannot explicitly handle recombination events or gene transfers, duplications at levels different from the gene, and propagation of insertion sequences. Some tools exist to reconstruct gene content or order in the presence of transfers [3,42], but not equivalent to ProfileNJ [36], which we used because of a lack of signal from the sequences in many gene families. It has not been developed for transfers apparently for algorithmic purposes [43]. Transfers will probably limit the quality of the sequence, which at recombination points will be reconstructed with a wrong gene tree. We expect these limits to be rare, as we found only little evidence of gene evolution clearly discordant with the species tree.

Another limit of this method is that it handles evolution at three different scales: sequence, gene content, gene order, while evolution happens at a continuum of scales, some part of it we don't explicitly model. This is for example the case for small duplications: gene duplications are handled but if they are smaller than genes, duplications will be part of sequence evolution, where the models and alignements take indels into account but not duplications. This is also the case of insertion sequence propagation. If insertion sequences are annotated as genes, their dynamics is sometimes so fast that parsimony duplication/loss principles are not accounting for it, even within a very small amount of time. If insertion sequences are taken in intergenic regions, they will again be handled inside alignments and yield a small amount of false positives.

A small part of the sequence is not reconstructed because of convergent rearrangements which have wiped the traces of some intergenic sequences. These convergent rearrangements also introduce one ambiguity in the ancestral gene order. It is possible that it reflects an ancestral polymorphism which has differently been resolved in different lineages.

Polymorphism, and the absence of it in our ancestral genome, is another limitation of such an approach. The ancient population was probably composed of several variants, and the 650 years might not be sufficient to sort out all of it. So we are not sure that a single organism carried the genome we reconstruct, but it might be a consensus of several genomes.

Yet these limits concern probably a very small percentage of the sequence, which is largely reconstructed with a total match to the ancient sequence. Beyond the methodological challenge and the interesting comparison with an ancient genome, the goal of such a reconstruction is not to find an application in synthetic biology, but to understand the evolution of this dangerous pathogen. Substitutions, which apparently are only a minor part of the story, are often the only marker of evolution (for example in [2]) because of a better availability of performing tools.

## Conclusions

In conclusion, we report here the reconstructed ancestral bacterial genome of an ancestral *Yersinia pestis*. The reconstruction is achieved using already published software and methods but also introduces methodological novelties, especially concerning ancestral adjacency graph linearization, leading to the obtention of larger reconstructed ancestral chromosome fragments.

The comparison of the reconstructed ancestral genome with an ancient sequence provides the opportunity to assess the quality of the reconstruction. It appears that while the reconstruction methods display some limits for events spanning more than a few nucleotides and smaller than a gene (for instance, a gene domain duplication), they yield good results concerning small (substitutions, short indels) and gene-scale events(for instance, gene duplications or rearrangements spanning at least a gene).

## Competing interests

The authors declare that they have no competing interests.

## Authors' contributions

WD, VD and ET conceived the method, WD implemented and tested it. WD and ET wrote the article.

## Supplementary Material

Additional File 1 DucheminDaubinTannier2015 supplementary file 2.fas. Fasta file containing the ancestral sequences obtained for the chromosome and plasmids of the ancestral *Yersinia pestis *species. Click here for file

Additional File 2 DucheminDaubinTannier2015 supplementary file 1.pdf. Histogram of the hit lengths (represented as its log10 here) when ancient contigs are aligned to the ancestral genome of *Yersinia pestis*. Click here for file
