# Supplementary material for: Reconstruction of an ancestral Yersinia pestis genome and comparison with an ancient sequence
Source: BMC Genomics. 2015 Oct 2;16(Suppl 10):S9. doi: 10.1186/1471-2164-16-S10-S9 (PMC4603589; doi:10.1186/1471-2164-16-S10-S9)

Fig S1. Histogram of the hit lengths (represented as its $\log_{10}$ here) when ancient contigs are aligned to the ancestral genome of *Yersinia pestis*.
The bimodality of the distribution is explained by the enrichment in insertion sequences and small repetitions of the *Yersinias* genomes, producing a large number of small hits, and led to the threshold of $10^{2.5}$ to exclude such hits.

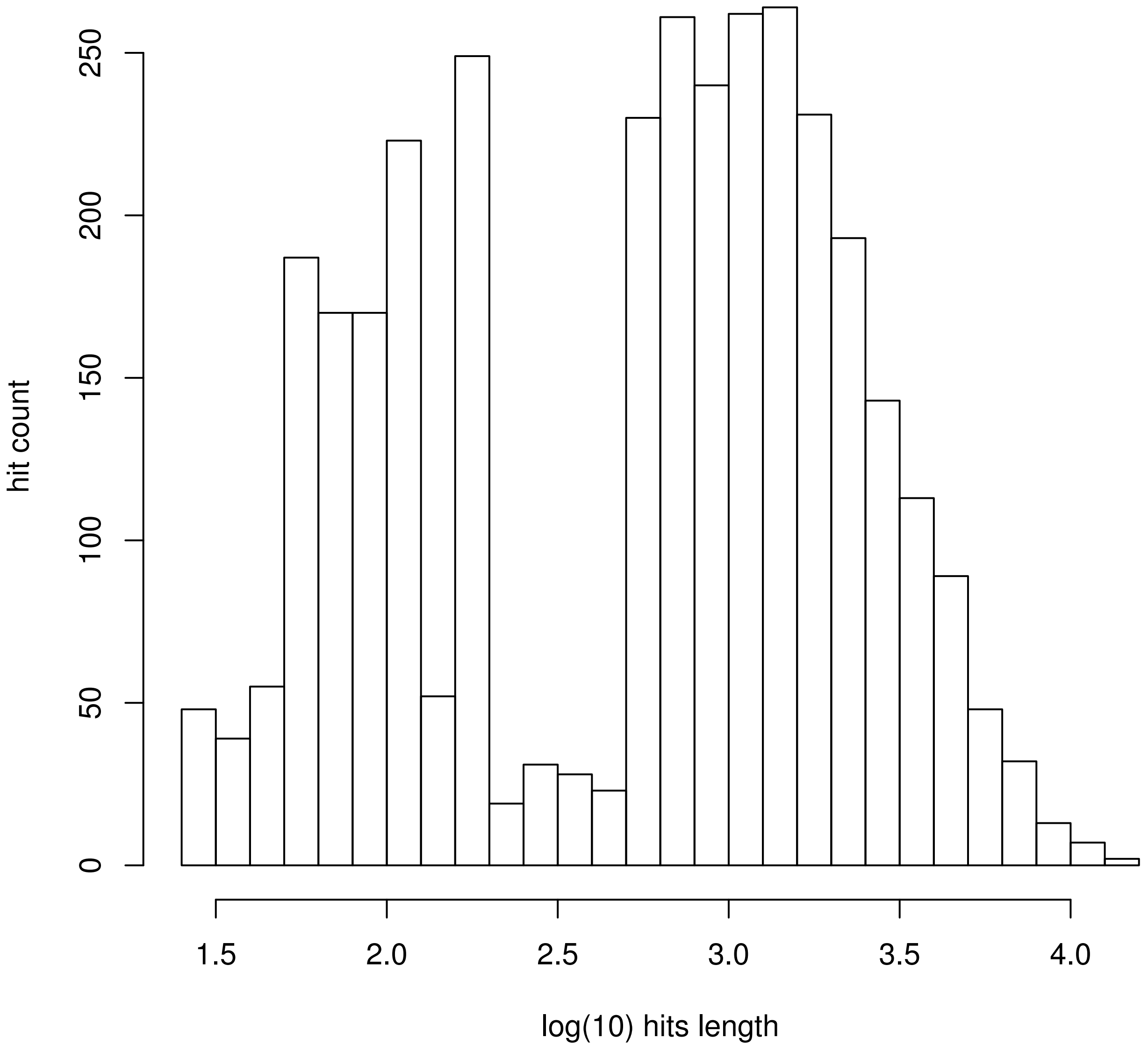

Supplement: Additional File 2 — DucheminDaubinTannier2015 supplementary file 1.pdf. Histogram of the hit lengths (represented as its log10 here) when ancient contigs are aligned to the ancestral genome of Yersinia pestis. [file 1471-2164-16-S10-S9-S2.pdf]
